# Endoscopic closure of an esophago-bronchial fistula after esophageal atresia repair using autologous adipose stromal vascular fraction and platelet-rich plasma injection

**DOI:** 10.1055/a-2846-4773

**Published:** 2026-04-20

**Authors:** Roos E. Pouw, Joris A. Van Dongen, Ludovica Magni, Arnold J. Bittermann, Sander Zwaveling, Michiel P. van Wijk, Stefaan H. A. J. Tytgat

**Affiliations:** 1 8124 Department of Gastroenterology and Hepatology, UMC Utrecht, Utrecht, The Netherlands; 2 8124 Department of Plastic-, Reconstructive- and Hand Surgery, UMC Utrecht, Utrecht, The Netherlands; 3 Department of Pediatric Surgery, Wilhelmina Childrenʼs Hospital, UTC Utrecht, Utrecht, The Netherlands; 4 Department of Pediatric Otorhinolaryngology, Wilhelmina Childrenʼs Hospital, UMC Utrecht, Utrecht, The Netherlands; 5 Department of Pediatric Surgery, Emma Children's Hospital, Amsterdam University Medical Center, Amsterdam, The Netherlands; 6 Department of Pediatric Gastroenterology, Emma Childrenʼs Hospital, Amsterdam University Medical Center, Location Vrije Universiteit, Amsterdam, The Netherlands; 7 Amsterdam Gastroenterology Endocrinology Metabolism and Amsterdam Reproduction and Development Research Institutes, Amsterdam UMC, VU University, Amsterdam, The Netherlands

Esophageal–airway fistulas are severe complications after esophageal atresia (EA) repair, occurring in 2–14% of patients and associated with chronic aspiration, recurrent respiratory infections, feeding difficulties, and failure to thrive. Endoscopic closure is less invasive but has limited success, whereas surgical revision is more effective yet carries substantial morbidity 1 . Regenerative therapies, including autologous adipose stromal vascular fraction (SVF) and platelet-rich plasma (PRP), have shown promising results in adult fistula treatment 2 3 .

A 16-month-old boy with long-gap type-C EA and tracheomalacia developed anastomotic leakage and a refractory stricture. Surgical revision was complicated by leakage and the formation of a cavernous esophagobronchial fistula to the right upper lung ( Fig. 1 **a** ). Endotracheal closure was not feasible, and endoscopic esophageal brush debridement was unsuccessful. Further surgery was considered undesirable, and the patient’s condition deteriorated with recurrent respiratory infections and severe failure to thrive.

**Fig. 1:**
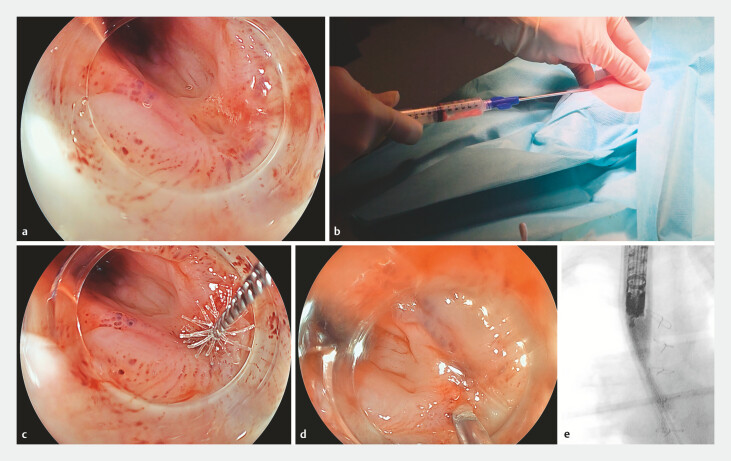
**a** During endoscopy the bronchoesophageal fistula is identified. **b** Adipose tissue is harvested from the upper legs. **c** The fistula is mechanically debrided using a cytology brush. **d** The SVF/PRP mixture is injected around the fistula in the submucosa. **e** Closure of the fistula was confirmed by contrast infusion of the esophagus after distal occlusion of the esophagus with a Fogarty catheter.

Endoscopic fistula closure using autologous SVF/PRP was performed ( Video 1 ). Adipose tissue was harvested ( Fig. 1 **b** ) and mechanically fractionated and centrifuged to obtain SVF (Arthrex ACP-SVF kit 4 ), and PRP was prepared from peripheral blood. After fistula identification and tract brushing ( Fig. 1 **c** ), SVF/PRP was injected circumferentially around the esophageal fistula orifice using a 7.9-mm endoscope (Fujifilm; Fig. 1 **d** ).

The video demonstrates the identification of the fistula; the preparation of the adipose stromal vascular fraction (SVF) and platelet rich plasma (PRP); the treatment of the fistula; and the mechanistical background information and endoscopic follow-up.Video 1

Following the procedure, respiratory symptoms resolved, infections ceased, and the child gained 1 kg. At 8 weeks, endoscopy with fluoroscopy confirmed fistula closure ( Fig. 1 **e** ).

SVF contains adipose stromal cells, endothelial progenitors, and immune cells, which exert anti-inflammatory and pro-regenerative effects. These effects are enhanced by the extracellular matrix, providing a perivascular niche for cell retention and sustained paracrine factor release. PRP further augments regenerative signaling. Mechanical shear stress during SVF isolation activates stromal cells toward a pro-regenerative phenotype, supporting SVF/PRP as a low-risk strategy for inoperable pediatric esophageal–airway fistulas 5 . To our knowledge, this represents the first reported infant successfully treated with endoscopic autologous adipose SVF/PRP for esophagobronchial fistula closure after EA repair.

Endoscopy_UCTN_Code_TTT_1AO_2AI

## References

[1] van StigtMJBHutJEReulingEMBPOutcome of recurrent tracheoesophageal fistula treatment after esophageal atresia repairJ Pediatr Surg20256016215939874825 10.1016/j.jpedsurg.2025.162159

[2] Boskoski I Pontecorvi V Caretto AA Endoscopic injection of autologous fat tissue for the treatment of chronic gastrointestinal fistulas Gut 2025gutjnl-2025–335754 10.1136/gutjnl-2025-335754 40592565

[3] SchoutenWRArkenboschJHCvan der WoudeCJEfficacy and safety of autologous adipose-derived stromal vascular fraction enriched with platelet-rich plasma in flap repair of transsphincteric cryptoglandular fistulasTech Coloproctol2021251301130934606026 10.1007/s10151-021-02524-6PMC8580893

[4] Van DongenJAGostelieOFEVonkLAFractionation of Adipose Tissue Procedure With a Disposable One-Hole FractionatorAesthet Surg J202040NP194NP20131402379 10.1093/asj/sjz223

[5] BanyardDASarantopoulosCNBorovikovaAAPhenotypic analysis of stromal vascular fraction after mechanical shear stress reveals stress-induced progenitor populationsPlast Reconstr Surg2016138237e247e10.1097/PRS.0000000000002356PMC584584127465185

